# Development, Evaluation, and Clinical Application of PRRSV-2 Vaccine-like Real-Time RT-PCR Assays

**DOI:** 10.3390/v15112240

**Published:** 2023-11-10

**Authors:** Gaurav Rawal, Karen M. Krueger, Wannarat Yim-im, Ganwu Li, Phillip C. Gauger, Marcelo N. Almeida, Ethan K. Aljets, Jianqiang Zhang

**Affiliations:** Department of Veterinary Diagnostic and Production Animal Medicine, College of Veterinary Medicine, Iowa State University, Ames, IA 50011, USA; grawal@iastate.edu (G.R.); kharmon@iastate.edu (K.M.K.); w.yimim@gmail.com (W.Y.-i.); liganwu@iastate.edu (G.L.); pcgauger@iastate.edu (P.C.G.); malmeida@iastate.edu (M.N.A.); ealjets@iastate.edu (E.K.A.)

**Keywords:** porcine reproductive and respiratory syndrome virus, PRRSV, vaccine-like, singleplex, multiplex, real-time RT-PCR, recombination

## Abstract

In this study, we developed and validated (1) singleplex real-time RT-PCR assays for specific detection of five PRRSV-2 MLV vaccine viruses (Ingelvac MLV, Ingelvac ATP, Fostera, Prime Pac, and Prevacent) and (2) a four-plex real-time RT-PCR assay (IngelvacMLV/Fostera/Prevacent/XIPC) including the internal positive control XIPC for detecting and distinguishing the three most commonly used vaccines in the USA (Prevacent, Ingelvac MLV, and Fostera). The singleplex and 4-plex vaccine-like PCRs and the reference PCR (VetMAX^TM^ PRRSV NA&EU, Thermo Fisher Scientific, Waltham, MA, USA) did not cross-react with non-PRRSV swine viral and bacterial pathogens. The limits of detection of vaccine-like PCRs ranged from 25 to 50 genomic copies/reactions. The vaccine-like PCRs all had excellent intra-assay and inter-assay repeatability. Based on the testing of 531 clinical samples and in comparison to the reference PCR, the diagnostic sensitivity, specificity, and agreement were in the respective range of 94.67–100%, 100%, and 97.78–100% for singleplex PCRs and 94.94–100%, 100%, and 97.78–100% for the 4-plex PCR, with a C_T_ cutoff of 37. In addition, 45 PRRSV-2 isolates representing different genetic lineages/sublineages were tested with the vaccine-like PCRs and the results were verified with sequencing. In summary, the vaccine-like PCRs specifically detect the respective vaccine-like viruses with comparable performances to the reference PCR, and the 4-plex PCR allows to simultaneously detect and differentiate the three most commonly used vaccine viruses in the same sample. PRRSV-2 vaccine-like PCRs provide an additional tool for detecting and characterizing PRRSV-2.

## 1. Introduction

Porcine reproductive and respiratory syndrome virus (PRRSV) is an enveloped, positive-sense, single-stranded RNA virus in the genus *Betaarterivirus*, family *Arteriviridae*, and order *Nidovirales*. According to the new virus taxonomy, PRRSV includes two species: *Betaarterivirus suid 1* (with virus name PRRSV-1) and *Betaarterivirus suid 2* (with virus name PRRSV-2) [1]. The PRRSV genome is approximately 15kb in length and includes ORF1a, ORF1b, ORF2a, ORF2b, ORF3, ORF4, ORF5a, ORF5, ORF6, ORF7, and a short transframe (TF) ORF in the nsp2 region [2,3]. The disease caused by this virus is called porcine reproductive and respiratory syndrome (PRRS), characterized by straddling clinical presentations, such as reproductive problems in breeding herds, respiratory issues in pigs of any age especially in the nursery and growing herds, and occasional neurological disorders, and causes tremendous economic losses [4,5].

Diagnostics, biosecurity, epidemiologic investigations, inducing immunity, and other strategies have been practiced over time to prevent and control PRRSV at the herd level. Vaccination with commercial modified live virus (MLV) vaccines is one important approach to inducing immunity to help control PRRSV [6,7]. PRRSV MLV vaccines have been shown to have varied protection against different PRRSV strains [7,8,9,10] and to be able to reduce production and economic losses and the shedding of wild-type viruses [11].

The first step for molecular detection of PRRSV is generally to test samples using a PRRSV screening PCR that targets the conserved genomic regions and can detect both wild-type and vaccine strains. However, the increasing use of PRRSV MLV vaccines presents challenges in interpreting the screening PCR-positive results, since the positive results could be due to the vaccine-like or wild-type PRRSV or recombinant virus. In order to determine whether the PRRSV detected by the screening PCR is a vaccine-like or a wild-type strain, ORF5 sequencing via the Sanger method and whole genome sequencing via next-generation sequencing (NGS) technology is generally conducted. Nevertheless, NGS is expensive and time-consuming and may be unsuccessful on samples with high C_T_ values [12]; ORF5 sequencing alone cannot rule out the presence of recombinant virus. PRRSV vaccine-like PCR assays can be an additional tool to help determine if a vaccine-like virus is present in a sample, and a combination of vaccine-like PCR assays, PRRSV screening PCR, and ORF5 sequencing can help to flag cases for further investigations.

Six commercial PRRSV-2 MLV vaccines (Ingelvac PRRS MLV, Ingelvac PRRS ATP, Fostera PRRS, Prime Pac PRRS, Prevacent PRRS, and PRRSGard) are available in the USA. In a proof-of-concept study, we developed a PRRSGard vaccine-like real-time RT-PCR assay with excellent performance based on the validation data [13]. In this study, the first objective was to develop and validate TaqMan probe-based singleplex real-time RT-PCR assays for the specific detection of the other five PRRSV-2 MLV vaccine viruses (Ingelvac PRRS MLV, Ingelvac PRRS ATP, Fostera PRRS, Prime Pac PRRS, and Prevacent PRRS). The second objective was to develop and evaluate a multiplex real-time RT-PCR, including an exogenous internal positive control (XIPC), for detecting and differentiating the three most commonly used vaccines in the USA (Prevacent PRRS, Ingelvac PRRS MLV, and Fostera PRRS). The multiplex PCR IngelvacMLV/Fostera/Prevacent/XIPC is referred to as 4-plex PCR in this study.

## 2. Materials and Methods

### 2.1. Primers and Probes

A total of eight different sets of primers and probes were tested in this study. The sequence information of forward and reverse primers and probes of singleplex and 4-plex PCRs included in this study are shown in Table 1. For Ingelvac PRRS MLV, a singleplex PCR targeting ORF5 gene (Ingelvac MLV Assay 1)—described in a conference proceeding [14]—and a singleplex PCR targeting nsp2 region (Ingelvac MLV Assay 2)—developed in this study—were included. Similarly, two singleplex PCR assays were included for Ingelvac PRRS ATP with Assay 1 targeting the ORF5 gene (as described in a conference proceeding [14]) and Assay 2 targeting nsp2 region (developed in a previous publication [15]). The singleplex PRRSGard PCR targeting the ORF1b/2 region was previously developed in our laboratory [13]. The remaining singleplex PCR assays for Fostera PRRS, Prevacent PRRS, and Prime Pac PRRS were developed in this study, and these three PCR assays all targeted the nsp2 region (Table 1).

For the four singleplex PCR assays developed in this study, we first aligned and compared the whole genome sequences of vaccine viruses and over 1000 PRRSV field and laboratory isolates and then designed the primers and probes targeting the genomic regions with unique sequences to each vaccine virus. After a thorough validation process, suitable singleplex PCR assays for Ingelvac MLV, Fostera, and Prevacent were selected to develop the 4-plex PCR, including the internal positive control XIPC. XIPC DNA is a fragment of nucleotides which are artificially designed and synthesized with a T7 promoter at the 5′ upstream. The XIPC sequence is not present in any analyzed pathogens or host species. XIPC DNA is in vitro transcribed into XIPC RNA, which is added to the extraction lysis buffer before nucleic acid extraction [16].

### 2.2. Nucleic Acid Extraction

Following the manufacturer’s instructions, nucleic acids were extracted using a MagMAX Pathogen RNA/DNA Kit (Thermo Fisher Scientific, Waltham, MA, USA) and a Kingfisher Flex instrument (Thermo Fisher Scientific). One hundred microliters of the sample was used for extraction, and nucleic acids were eluted into 90 µL of elution buffer. Before nucleic acid extraction, an internal positive control XIPC RNA (1 × 10^4^ copies per extraction) was added to the extraction lysis buffer. Thus, the extracted nucleic acids from each sample were expected to contain both the XIPC RNA and the target pathogen nucleic acid [16].

### 2.3. Commercial PRRSV Screening PCR

A commercial PRRSV screening real-time RT-PCR, VetMAX^TM^ PRRSV NA&EU Reagent v2 (Thermo Fisher Scientific), which can simultaneously detect and differentiate PRRSV-1 and PRRSV-2, was included in this study as a reference PCR. This reference PCR targets the conserved genomic regions in the PRRSV genome and can detect both wild-type and vaccine-like PRRSV strains. The information on primers and probes of the commercial VetMAX™ PRRSV NA&EU Reagent is unavailable. The reference PCR reaction setup, thermal cycling conditions, and the settings for analysis have been previously described [13]. All the vaccine-like PCRs validated in this study were compared with this reference PCR.

### 2.4. Singleplex PRRSV-2 Vaccine-like PCRs

For the singleplex Ingelvac PRRS MLV PCR Assay 1 and Assay 2, Ingelvac PRRS ATP PCR Assay 1 and Assay 2, Fostera PRRS PCR, Prevacent PRRS PCR, Prime Pac PRRS PCR, and PRRSGard PCR, the final concentration of 400 nM was used for each vaccine virus primer and 200 nM was used for each vaccine probe. In brief, each PCR was set up in a 20 µL reaction: 5 µL of TaqMan^®^ Fast 1-Step Master Mix (Thermo Fisher Scientific), 0.4 µL of vaccine forward primer at 20 µM, 0.4 µL of vaccine reverse primer at 20 µM, 0.4 µL of vaccine probe at 10 µM, 0.2 µL XIPC forward primer at 20 µM, 0.2 µL of XIPC reverse primer at 20 µM, 0.15 µL of XIPC probe at 10 µM, 8.25 µL nuclease-free water, and 5 µL nucleic acid extract. Amplification reactions were performed on an ABI 7500 Fast Instrument (Thermo Fisher Scientific) with the following PCR conditions: one cycle of 50 °C for 5 min, one cycle of 95 °C for 20 s, and 40 cycles of 95 °C for 3 s and 60 °C for 30 s. The analysis was performed using an automatic baseline, a vaccine probe detector at a threshold of 0.1, and an XIPC detector (Cy5) at 10% of the maximum height of the sigmoid amplification curve.

### 2.5. PRRSV-2 IngelvacMLV/Fostera/Prevacent/XIPC 4-Plex PCR

The 4-plex PCR was set up using TaqMan^®^ Fast 1-Step Master Mix (Thermo Fisher Scientific). Ingelvac MLV, Fostera, Prevacent, and XIPC probes were labeled with the fluorescence dyes ABY, VIC, FAM, and Cy5, respectively. The concentrations of each virus primer in the 4-plex PCR were optimized at 150 nM, 300 nM, 400 nM, 600 nM, and 900 nM and compared to the commercial PRRSV screening reference PCR using serially diluted vaccine viruses. Eventually, the optimized 4-plex PCR was set up in a 20 µL reaction including the following components: (1) 5 µL of TaqMan^®^ Fast 1-Step Master Mix; (2) 0.6 µL of Ingelvac MLV forward primer at 20 µM (final concentration 600 nM), 0.6 µL of Ingelvac MLV reverse primer at 20 µM (final concentration 600 nM), and 0.4 µL of Ingelvac MLV probe at 10 µM (final concentration 200 nM); (3) 0.6 µL of Fostera forward primer at 20 µM (final concentration 600 nM), 0.6 µL of Fostera reverse primer at 20 µM (final concentration 600 nM), and 0.4 µL of Fostera probe at 10 µM (final concentration 200 nM); (4) 0.3 µL of Prevacent forward primer at 20 µM (final concentration 300 nM), 0.3 µL of Prevacent reverse primer at 20 µM (final concentration 300 nM), and 0.4 µL of Prevacent probe at 10 µM (final concentration 200 nM); (5) 0.2 µL of XIPC forward primer at 20 µM (final concentration 200 nM), 0.2 µL of XIPC reverse primer at 20 µM (final concentration 200 nM), and 0.15 µL of XIPC probe at 10 µM (final concentration 75 nM); (6) 5.25 µL nuclease-free water; (7) 5 µL nucleic acid extract.

Amplification reactions were performed on an ABI 7500 Fast instrument (Thermo Fisher Scientific) with the following conditions: one cycle of 50 °C for 5 min, one cycle of 95 °C for 20 s, and 40 cycles of 95 °C for 3 s and 60 °C for 30 s. The analysis was performed using 7500 Fast System SDS Software version 1.5.1 (Thermo Fisher Scientific) with an automatic baseline, Ingelvac MLV detector (ABY) at a threshold of 10%, Fostera detector (VIC) at a threshold of 10%, Prevacent detector (FAM) at a threshold of 5%, and XIPC detector (Cy5) at a threshold of 10% of the sigmoid amplification curve’s maximum height, respectively.

### 2.6. Analytical Specificity

The analytical specificities of the singleplex and 4-plex vaccine-like PCRs were evaluated using 27 non-PRRSV swine viral and bacterial pathogens, 6 commercial PRRSV-2 MLV vaccine viruses, 1 PRRSV-1 isolate, and 45 PRRSV-2 laboratory and field isolates representing the major ORF5-based genetic lineages/sublineages of PRRSV circulating in the United States. A commercial real-time PRRSV screening PCR (VetMAX^TM^ PRRSV NA&EU, Thermo Fisher Scientific) was used as a reference PCR for comparison. Mixtures of different vaccine viruses at equal volume ratios were also tested to examine the analytical specificity of the 4-plex PCR.

### 2.7. Analytical Sensitivity

For analytical sensitivity analysis, serial dilutions (3 replicates per dilution) of each vaccine virus with known infectious titers were tested with the corresponding vaccine-like singleplex PCR, 4-plex PCR, and the commercial PRRSV screening reference PCR.

### 2.8. In Vitro Transcribed RNA

For each of the vaccine viruses Ingelvac MLV, Ingelvac ATP, Fostera, Prevacent, and Prime Pac, a double-stranded and linear gBlock DNA fragment of different nucleotide (nt) lengths (729 nt, 737 nt, 719 nt, 723 nt, and 738 nt, respectively) containing the nsp2 genomic region of an individual vaccine virus with a T7 promoter at the upstream region was synthesized (Integrated DNA Technologies, Coralville, IA, USA) with a molecular weight of 450,346.3; 455,287.5; 444,167.3; 446,657.6; and 455,912.8, respectively, and an amount of 500 ng for each gBlock DNA.

The gBlock DNA fragment was subjected to run-off in vitro transcription into RNA using a MEGAscript^®^ Kit (Thermo Fisher Scientific) following the manufacturer’s instructions. RNA transcripts were produced, treated with DNase I, and purified with MEGAclear™ Transcription Clean-up kit (Thermo Fisher Scientific) using the lithium chloride precipitation method and following the manufacturer’s instructions. Copy numbers of RNA transcripts were calculated based on concentrations determined using a Qubit 4 Fluorometer (Thermo Fisher Scientific). Serial dilutions of in vitro transcribed (IVT) RNAs were prepared using nucleic acid dilution solution (Thermo Fisher Scientific). Aliquots were frozen at −80 °C for single use of each aliquot.

### 2.9. Limit of Detection of Singleplex and IngelvacMLV/Fostera/Prevacent/XIPC 4-Plex Vaccine-like PCRs

Serial dilutions of Ingelvac MLV, Ingelvac ATP, Fostera, Prevacent, and Prime Pac IVT RNA were tested with singleplex vaccine-like PCRs, and the serial dilutions of IVT RNAs of Ingelvac MLV, Fostera, and Prevacent were tested with the 4-plex PCR with 3 replicates at high concentrations and 20 replicates at low concentrations for each dilution. The highest dilution that provided positive results in at least 95% of reactions was considered the limit of detection for that PCR assay.

### 2.10. Repeatability of Singleplex and 4-Plex Vaccine-like PCRs

To assess the repeatability of the singleplex and 4-plex vaccine-like PCRs, each dilution of the respective vaccine virus IVT RNA was tested in triplicate in the same plate for intra-assay repeatability, and tested in 3 different PCR plates for inter-assay repeatability. Generally, intra-assay repeatability of less than 10% and inter-assay repeatability of less than 15% is acceptable. For each singleplex vaccine-like PCR and the 4-plex PCR, the repeatability coefficient of variation (CV) was calculated using the formula where the PCR C_T_ value standard deviation is divided by the mean of PCR C_T_ value at each concentration of IVT RNA.

### 2.11. Clinical Samples and Diagnostic Performances of Singleplex and 4-Plex Vaccine-like PCRs

Serum samples collected from pigs that were experimentally vaccinated with each vaccine virus were used to evaluate the diagnostic performances of singleplex and 4-plex vaccine-like PCRs. The experimental protocol of this study on pigs was approved by the Iowa State University Institutional Animal Care and Use Committee (IACUC-22-016) and the Institutional Biosafety Committee (IBC-22-014). Briefly, 70 pigs at 3–4 weeks of age were divided into 6 groups (10 pigs/group). Pigs in 6 groups were vaccinated with Ingelvac PRRS MLV, Ingelvac PRRS ATP, Fostera PRRS, Prevacent PRRS, Prime Pac PRRS, and virus-negative culture medium, respectively, via intramuscular injection following the doses recommended by vaccine manufacturers. For the Ingelvac PRRS ATP group, one pig died due to bleeding at 10 days post vaccination (DPV) and hence was removed from the analysis. Serum samples collected at 0, 2, 4, 7, 10, 14, 21, 28, and 35 DPV were tested with the vaccine-specific PCRs and the reference PCR. Specifically, serum samples from 10 mock-vaccinated pigs (n = 90) and 10 pigs vaccinated with Ingelvac MLV (n = 90) were tested with the Ingelvac MLV singleplex PCR Assay 2 and the 4-plex PCR. Serum samples from 10 mock-vaccinated pigs (n = 90) and 9 Ingelvac ATP-vaccinated pigs (n = 81) were tested with the Ingelvac ATP singleplex PCR Assay 2. Serum samples from 10 mock-vaccinated pigs (n = 90) and 10 Fostera-vaccinated pigs (n = 90) were tested with the Fostera singleplex PCR and the 4-plex PCR. Serum samples from 10 mock-vaccinated pigs (n = 90) and 10 Prevacent-vaccinated pigs (n = 90) were tested with the Prevacent singleplex PCR and the 4-plex PCR. Serum samples from 10 mock-vaccinated pigs (n = 90) and 10 pigs vaccinated with Prime Pac (n = 90) were tested with the Prime Pac singleplex PCR.

The diagnostic sensitivity was calculated using the following formula: positive clinical samples of both PCR assays (i.e. individual vaccine-specific PCR and reference PCR) were divided by total positive samples determined with the reference PCR. The diagnostic specificity was calculated using the formula where negative clinical samples of both PCR assays were divided by total negative samples determined with the reference PCR. The agreement or accuracy was measured using the following formula: the sum of positive clinical samples of both PCR assays and negative clinical samples of both PCR assays is divided by the total number of samples tested.

### 2.12. Performance of 4-Plex PCR on Samples Containing a Mixture of Vaccine Virus and a Wild-Type PRRSV Isolate

To determine whether the 4-plex PCR can detect a vaccine virus in a sample co-infected with a wild-type PRRSV strain, the Ingelvac MLV, Fostera, and Prevacent vaccine viruses were each manually mixed with a PRRSV wild-type field isolate USA/IN/65239GA/2014 (RFLP 1-7-4; sublineage L1A) at different concentration ratios (1:1, 10^−1^:1, 10^−2^:1, 10^−3^:1, 10^−4^:1, 10^−5^:1, 10^−6^:1, and 10^−7^:1) followed by testing with the 4-plex PCR and the reference PCR. For the wild-type isolate, the same volume and the same stock was used. For the vaccine viruses, each vaccine virus was 10-fold serially diluted (10^0^ to 10^−7^) and then mixed with the wild-type virus at an equal volume.

### 2.13. Sequencing to Confirm Virus Identity

The ORF5 sequences of the 45 PRRSV-2 field and laboratory isolates, obtained in either MARC-145 or ZMAC cells, were determined with the Sanger method following the previously described procedures [17] to confirm the vaccine-like PCR results. The ORF5-based genetic lineages and sublineages were further determined for these 45 PRRSV-2 isolates following the recently described genetic classification system [18]. Thirteen selected PRRSV-2 isolates (#10, #11, #12, #13, #14, #16, #26, #27, #28, #32, #33, #37, and #42) were subjected to whole genome sequencing via NGS following the previously described procedures [12]. The whole genome sequences of these thirteen isolates were deposited into GenBank with the accession numbers of OR669515–OR669527. The whole genome sequences of three other isolates were previously deposited into GenBank, and they were also included in this study for analysis: isolate #17 (GenBank accession number EF488739), isolate #23 (ON100576), and isolate #36 (MK796165).

To identify if recombination was present in the virus isolates of interest, the whole genome sequences of such isolates were analyzed against the six PRRSV-2 vaccine viruses and the PRRSV-2 whole genome sequences available in GenBank or our database. Recombination screening in the multiple sequence alignments of complete genome sequences was performed using Recombination Detection Program v5.43 (RDP5) [19]. Potential recombination events detected in RDP5 were confirmed using a window size of 200 and a step size of 20 bp in SimPlot v3.5.1 [20].

## 3. Results

### 3.1. Analytical Specificity of the Singleplex and 4-Plex Vaccine-like PCRs

All of the singleplex PCRs did not cross-react with PRRSV-1 (Lelystad strain) and any of the tested non-PRRSV swine viruses and bacteria (Table 2). The singleplex Ingelvac MLV Assay 1 and Ingelvac ATP Assay 1 cross-reacted with Prevacent and Fostera vaccine virus, respectively, and were excluded from further validation (Table 2). Notably, the Prime Pac PCR reacted with both the Prime Pac vaccine virus and PRRSGard vaccine virus, whereas the PRRSGard PCR only reacted with the PRRSGard vaccine virus and did not cross-react with the Prime Pac vaccine virus. Ingelvac MLV PCR Assay 2, Ingelvac ATP PCR Assay 2, Fostera PCR, and Prevacent PCR did not cross-react with any other MLV vaccine viruses, and all of them, together with Prime Pac PCR, were selected for further validation. In addition, the singleplex Ingelvac MLV PCR Assay 2, Fostera PCR, and Prevacent PCR were selected, together with the XIPC PCR, to develop the IngelvacMLV/Fostera/Prevacent/XIPC 4-plex PCR, followed by validation.

**Figure 1:**
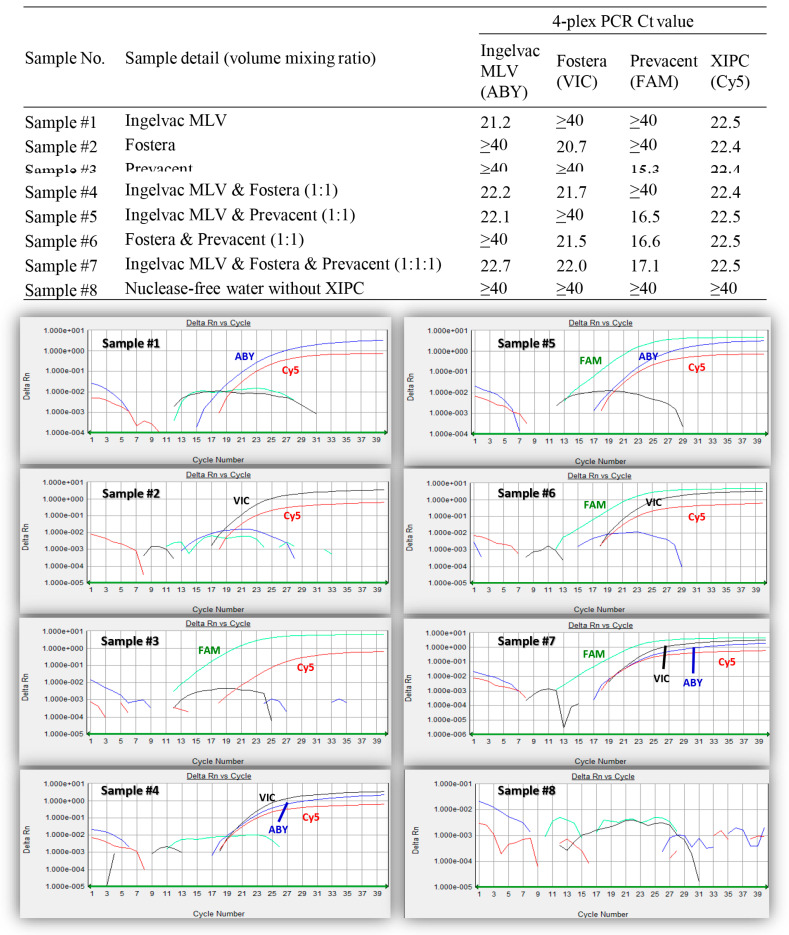
Different mixtures of Ingelvac MLV, Fostera, and Prevacent viruses tested with the IngelvacMLV/Fostera/Prevacent/XIPC 4-plex PCR. The identities of the samples #1–#8 are denoted at the top. Amplification plots of the 8 samples tested with IngelvacMLV/Fostera/Prevacent/XIPC 4-plex PCR are shown at the bottom. The reporter dyes ABY, VIC, FAM, and Cy5 are shown in blue, black, green, and red color, respectively.

As shown in Table 2, the 4-plex PCR specifically recognized Ingelvac MLV, Fostera, Prevacent, and XIPC with the fluorescence dyes ABY, VIC, FAM, and Cy5, respectively, and did not cross-react with other vaccine viruses, non-PRRSV swine viruses, or bacteria. The 4-plex PCR could also simultaneously detect and distinguish the Ingelvac MLV, Fostera, and Prevacent vaccine viruses when these three vaccine viruses were manually mixed in different combinations, as shown in Figure 1.

### 3.2. Analytical Sensitivity of the Singleplex and 4-Plex Vaccine-like PCRs

When the analytical sensitivities of the PCR assays were determined using serial dilutions of each vaccine virus and the detection endpoints were compared, the Ingelvac MLV PCR Assay 2, the 4-plex PCR for detecting Ingelvac MLV, the Ingelvac ATP PCR Assay 2, the Prevacent PCR assay, and the 4-Plex PCR for detecting Prevacent were 10-fold less sensitive than the reference screening PCR; the Fostera PCR assay, the 4-plex PCR for detecting Fostera, and the Prime Pac PCR assay were similar to the reference screening PCR, as shown in Table 3.

### 3.3. Limit of Detection of the Singleplex and 4-Plex Vaccine-like PCRs Using Respective Vaccine IVT RNA

The limit of detection (at least 95% of reactions were positive) was 50 genomic copies/reactions for the Ingelvac MLV PCR Assay 2, Ingelvac ATP PCR Assay 2, Fostera PCR assay, Prime Pac PCR assay, and the 4-plex PCR for detecting Ingelvac MLV and Fostera, and 25 genomic copies/reactions for Prevacent PCR assay and the 4-plex PCR for detecting the Prevacent vaccine virus under the conditions of this study, as shown in Table 4.

### 3.4. Repeatability of Singleplex and 4-Plex Vaccine-like PCRs

The singleplex PCRs had excellent intra-assay and inter-assay repeatability with the average intra-assay coefficient of variation (CV) of only 1.22% and 0.76% for Ingelvac MLV, 1.44% and 1.60% for Ingelvac ATP, 0.63% and 0.96% for Fostera, 0.95% and 0.75% for Prevacent, and 0.76% and 0.98% for Prime Pac, as shown in Table 5. Likewise, the 4-plex PCR also had outstanding intra-assay and inter-assay repeatability with the average intra-assay CV of only 1.33% and 1.30% for Ingelvac MLV, 1.05% and 1.11% for Fostera, and 1.29% and 0.68% for Prevacent, as shown in Table 5.

### 3.5. Diagnostic Performance of the Singleplex and 4-Plex Vaccine-like PCRs on Clinical Samples

Based on the testing of clinical samples and the comparison to the reference screening PCR (a C_T_ cutoff of 37 was used for all singleplex vaccine-like PCRs and the reference screening PCR), the diagnostic sensitivity, specificity, and agreement were 98.48%, 100%, and 99.44%, respectively, for the Ingelvac MLV PCR Assay 2; 98.44%, 100%, and 99.42% for the Ingelvac ATP PCR Assay 2; 94.94%, 100%, and 97.78% for the Fostera PCR assay; 100%, 100%, and 100% for the Prevacent PCR assay; and 94.67%, 100%, and 97.78% for the Prime Pac PCR assay. Compared to the reference screening PCR, the diagnostic sensitivity, specificity, and agreement of the 4-plex PCR were 98.48%, 100%, and 99.44% for detecting Ingelvac MLV, 94.94%, 100%, and 97.78% for detecting Fostera, and 100%, 100%, and 100% for detecting the Prevacent vaccine virus when a C_T_ cutoff of 37 was used for all PCR assays, as shown in Table 6.

The 10 serum samples having discrepancies between the reference PCR and vaccine-specific PCRs all had relatively high C_T_ values ranging from 34.9 to 36.9 (Table 7). Due to low concentrations (high C_T_ values) of the vaccine virus in these samples, ORF5 sequencing via the Sanger method was performed to confirm the virus identity but sequencing was unsuccessful.

### 3.6. Performance of the 4-Plex PCR Determined with Manual Mixture of Vaccine Virus and a Wild-Type PRRSV Isolate

The wild-type PRRSV isolate USA/IN/65239GA/2014 stock had a C_T_ of 16.1 with the reference PCR and was negative with the 4-plex PCR (Table 8). The Ingelvac MLV, Fostera, and Prevacent vaccine virus stock had a C_T_ of 18.1, 16.6, and 15.1 with the reference PCR, and a C_T_ of 21.6, 20.9, and 15.0 with the 4-plex PCR, respectively (Table 8). The mixtures 1 to 8 contained a similar concentration of the wild-type virus and decreasing concentration of the Ingelvac MLV vaccine virus. Consequently, the mixtures 1 to 8 all had similar C_T_ values with the reference PCR (C_T_ 16.8–17.9), but had increasing C_T_ values for the Ingelvac MLV vaccine virus with the 4-plex PCR. A similar pattern of results was obtained for the mixtures of the Fostera vaccine virus with the wild-type virus and the mixtures of the Prevacent vaccine virus with the wild-type virus (Table 8). The results clearly indicated that the 4-plex PCR can specifically detect the respective vaccine virus in a sample, even if a wild-type PRRSV strain was present in the sample. It was possible to reveal whether a vaccine virus and another virus strain were co-present in a sample by considering the C_T_ values of both vaccine-like PCRs and the PRRSV screening PCR.

### 3.7. Performance of the Singleplex and 4-Plex Vaccine-like PCRs on PRRSV-2 Isolates Representing Different Genetic Lineages and Sublineages

In order to determine whether the singleplex and the 4-plex vaccine-like PCRs can correctly detect the respective vaccine-like virus among the genetically diverse PRRSV isolates, we included 45 PRRSV-2 field and laboratory isolates for evaluation in this study (Table 9). These 45 isolates represented the major genetic lineages and sublineages of PRRSV-2 viruses currently circulating in North America, and they belonged to L1A, L1B, L1C, L1C.1, L1C.5, L1D, L1E, L1F, L1H, L5A, L8A, L8B, L8C, L8D, L9A, and L9. The ORF5-based RFLP patterns and the ORF5 nucleotide identities of these isolates to each of the six PRRSV-2 vaccines are summarized in Table 9. These 45 isolates were tested with the singleplex vaccine-like PCRs (Ingelvac MLV Assay 2, Ingelvac ATP Assay 2, Fostera, Prevacent, Prime Pac, and PRRSGard), the 4-plex PCR, and the PRRSV screening PCR (reference PCR); the results are summarized in Table 10.

All 45 isolates were identified as positive with the PRRSV screening PCR, with C_T_ values listed in Table 10. For most of the PRRSV-2 isolates whose ORF5 sequences were distantly related to vaccine viruses, they were negative with the respective vaccine-like PCR (isolates #1–9, #15, #21–22, #34, and #43–45), with consistent results between vaccine-like PCRs and ORF5 sequencing (Table 9 and Table 10). The remaining isolates are discussed in detail below.

The isolates #23–28 (all L5A) had 94.2–99.5% ORF5 nucleotide identities to the Ingelvac MLV vaccine virus and were all positive with the Ingelvac MLV PCR Assay 2 and the 4-plex PCR for the Ingelvac MLV virus (Table 9 and Table 10). In order to corroborate the results at the whole genome level, isolates #23 and #26–28 were tested with NGS. The whole genome sequence of isolate #23 had a 99.84% nucleotide identity to the Ingelvac PRRS MLV vaccine virus, confirming that this isolate was an Ingelvac MLV vaccine-like virus, and the Ingelvac MLV vaccine-like PCR result was correct. For isolates #26, #27, and #28, a consensus whole genome sequence was obtained from each of them, and the sequences respectively had 98.17%, 98.61%, and 96.73% nucleotide identity to the Ingelvac MLV vaccine virus at the whole genome level, suggesting that these three isolates were Ingelvac MLV vaccine-like or vaccine-derived viruses, which is consistent with the Ingelvac MLV vaccine PCR results. Notably, isolate #28 appeared to be a wild-type virus based on the ORF5 sequence (94.2% nt identity to the Ingelvac MLV vaccine virus); however, the whole genome sequence indicated that it may be a vaccine-derived virus. Interestingly, for isolates #26 and #27, there were large C_T_ differences between the screening PCR and Ingelvac MLV PCR (10.3 vs. 21.2 for isolate #26 and 15.8 vs. 25.7 for isolate #27), implying that there may be co-infection with multiple PRRSV strains in each of these two samples. Single nucleotide variation analysis on the NGS read data confirmed that isolates #26 and #27 contained not only an Ingelvac MLV vaccine-like sequence but also some non-vaccine sequences.

Isolates #29–33 (all L8A) had 96.8–100% ORF5 nucleotide identities to the Ingelvac ATP vaccine virus and were all positive with the Ingelvac ATP PCR Assay 2, except isolate #32, which was negative with the Ingelvac ATP PCR Assay 2 (Table 9 and Table 10). In order to help interpret the results at the whole genome level, isolates #32 and #33 were tested with NGS. The whole genome sequence of isolate #32 had a 98.50% nucleotide identity to the Ingelvac ATP vaccine virus, suggesting that this isolate was an Ingelvac ATP vaccine-like virus, and the Ingelvac ATP PCR Assay 2 result was incorrect. Comparison of the Ingelvac ATP PCR Assay 2 primer and probe sequences with the isolate #32 sequence indicated that there are three nucleotide mismatches in the forward primer and two nucleotide mismatches in the reverse primer. The sequence and PCR results on isolate #32 suggest that the Ingelvac ATP PCR Assay 2 is not perfect, and further improvement in designing the primers is needed. A consensus whole genome sequence of isolate #33 was obtained, and it had a 97.2% nucleotide identity to the Ingelvac ATP vaccine virus at the whole genome level, suggesting that this isolate was an Ingelvac ATP vaccine-like or vaccine-derived virus, which is consistent with the Ingelvac ATP PCR Assay 2 result. Interestingly, for isolate #33, there was a large C_T_ difference (11.9 vs. 22.2) between the screening PCR and Ingelvac ATP PCR, implying that there may be a co-infection with multiple PRRSV strains in this sample. Single nucleotide variation analysis on the NGS read data confirmed that isolate #33 contained not only an Ingelvac ATP vaccine-like sequence but also some non-vaccine sequences.

Isolates #35–42 (all L8C) had 95.4–99.7% ORF5 nucleotide identities to the Fostera vaccine virus and they (except isolates #36 and #37) were positive with the singleplex Fostera PCR and the 4-plex PCR for the Fostera virus (Table 9 and Table 10). Isolate #14 had an 87.7% ORF5 nucleotide identity to the Fostera vaccine virus, but it was positive with the singleplex Fostera PCR and the 4-plex PCR for the Fostera virus (Table 9 and Table 10). In order to help interpret the results at the whole genome level, isolates #36, #37, #42, and #14 were tested with NGS. The analyses of the whole genome sequences of isolates #36, #37, and #14 suggested that these three isolates were recombinant viruses (Table 11). For isolate #36, the recombination breakpoint was around the nucleotide position 6178; the nucleotides before the breakpoint were wild-type sequences, whereas the nucleotides after the breakpoint were Fostera vaccine-like. This explained why the ORF5 sequence suggested that isolate #36 was Fostera vaccine-like while Fostera PCR targeting the nsp2 region was negative. For isolate #37, recombination at three breakpoints (at positions 4854, 9075, and 10,542) appeared to occur: (1) the nucleotides 1–4853 were wild-type sequences; (2) the nucleotides 4854–9074 were Fostera vaccine-like; (3) the nucleotides 9075–10,541 were wild-type sequences; and (4) the nucleotides 10,542–15,011 were likely Fostera vaccine-derived. This explained why the ORF5 sequence suggested that isolate #37 was Fostera vaccine-like while the Fostera PCR targeting the nsp2 region was negative. Isolate #14 appeared to have two recombination breakpoints (at positions 685 and 12,190). Nucleotides 1–684 and 12,190–15,386 were wild-type sequences, whereas nucleotides 685–12,189 were likely Fostera vaccine-derived. This explained why the ORF5 sequence suggested that isolate #14 was a wild-type virus while the Fostera PCR targeting the nsp2 region was positive. For isolate #42, a consensus whole genome sequence was obtained, and it had a 97.9% nucleotide identity to the Fostera vaccine virus, suggesting that this isolate was a Fostera vaccine-like or vaccine-derived virus, which is consistent with the Fostera PCR result. For isolate #42, although there was a C_T_ difference (16.9 vs. 25.72) between the screening PCR and Fostera PCR, single nucleotide variation analysis on the NGS read data did not reveal the presence of multiple PRRSV strains.

Isolates #10–13 had 94.5–99.3% ORF5 nucleotide identities to the Prevacent vaccine virus; three of them (#10, #11, and #13) were positive, and one isolate (#12) was negative with the singleplex Prevacent PCR and the 4-plex PCR for the Prevacent virus (Table 9 and Table 10). In order to help interpret the results, these four isolates were subjected to NGS testing. Isolates #10 and #11 respectively had 98.6% and 99.3% nucleotide identity to the Prevacent vaccine virus at the whole genome level, and the Prevacent PCR results were consistent with the sequencing results. For isolate #12, its whole genome sequence had an 84.5% nucleotide identity, although its ORF5 sequence had a 96.7% nucleotide identity, to the Prevacent vaccine virus. Sequence analysis indicated that isolate #12 was a recombinant virus with a breakpoint around position 13,486; the nucleotides before the breakpoint were wild-type viruses, whereas the nucleotides after the breakpoint were similar to the Prevacent vaccine virus (Table 11). This explained why the Prevacent PCR targeting to the nsp2 region was negative on isolate #12. Isolate #13 had 94.0% and 94.5% nucleotide identity to the Prevacent vaccine virus at the whole genome and the ORF5 level, respectively. Isolate #13 appeared to be a wild-type virus, although it was positive with the Prevacent PCR.

Isolates #16–20 had 94.5–100% ORF5 nucleotide identities to the PRRSGard vaccine virus, but were all were negative with the PRRSGard PCR. As previously reported, the PRRSGard vaccine virus is a chimeric virus with structural protein genes similar to MN184-like wild-type viruses [13]. The ORF5 sequence itself cannot accurately tell whether an isolate is a PRRSGard vaccine-like virus or not; in contrast, the PRRSGard PCR targeting the unique genetic marker in the nsp12-ORF2 region can specifically detect the PRRSGard vaccine-like virus [13]. Whole genome sequences were determined on isolates #16 and #17, and they respectively had 85.8% and 88.5% nucleotide identity at the whole genome level to the PRRSGard vaccine virus, confirming that these two isolates were wild-type viruses and that the negative PRRSGard PCR results were correct.

None of the 45 isolates had similar ORF5 sequences to the Prime Pac vaccine virus. For those isolates (#10–14, #16–17, #23, #26–28, #32–33, #36–37, and #42) whose whole genome sequences were determined, none of them were similar to the Prime Pac vaccine virus, based on the whole genome sequence analysis.

## 4. Discussion

Different assays are used to detect and diagnose PRRSV infection, including PCR, sequencing, virus isolation, gross and microscopic lesion examination, immunohistochemistry, and antibody testing [4]. Among them, the real-time RT-PCR is the most commonly used tool in PRRSV detection because of its excellent sensitivity, specificity, high throughput capability, and short test turnaround time [22]. Multiplex PCR allows the detection of multiple agents in the same sample [23,24]. The common structure for detecting PRRSV is to first test samples using PRRSV screening RT-PCR that targets the conserved genomic regions and can detect both wild-type and vaccine strains without differentiating them. Distinguishing whether the detected virus is a wild-type or vaccine strain helps to prevent the movement and transmission of pigs unknowingly infected with a wild-type PRRSV. Additionally, this differentiation aids in monitoring vaccine virus shedding, implementing effective herd management practices, identifying potential breaches in the biosecurity procedures, and gaining insights into the current PRRSV status of the farm.

In 2014, a research group described the development of real-time RT-PCRs targeting the ORF5 gene for a specific detection of Ingelvac PRRS MLV (FAM dye) and Ingelvac PRRS ATP (HEX dye) vaccine viruses in a conference proceeding [14]. However, those two RT-PCRs cross-reacted with some vaccine viruses and multiple wild-type PRRSV strains when tested in our lab. A group in China established a multiplex PCR targeting the nsp2 region for a differential detection of PRRSV-2 classical strains, HP-PRRSV, and TJM-F92 vaccine strain derived from an HP-PRRSV isolate TJ [25]. Another group in China developed a multiplex PCR targeting the nsp2 and ORF4–ORF5 region for detecting and differentiating PRRSV-2 classical strains, HP-PRRSV, and JXA1-R vaccine strain derived from an HP-PRRSV isolate JXA1 [26]. As a proof-of-concept study, we designed primers and probe targeting the unique nsp12–ORF2 region and developed a PRRSGard vaccine-like PCR with excellent performance [13]. Recently, a research group in the USA developed a bead-based Luminex assay for the detection and differentiation of four PRRSV-2 vaccines (Ingelvac PRRS MLV, Ingelvac PRRS ATP, Fostera PRRS, and Prime Pac PRRS) and field strains of PRRSV [15]. However, the analytical sensitivity of the Luminex assay was one to two log_10_ lower than that of the real-time RT-PCR assays [15]. New and better real-time RT-PCR assays for detecting PRRSV vaccine-like viruses were needed.

In this study, we developed and evaluated singleplex real-time RT-PCRs targeting the nsp2 regions for detecting the Ingelvac PRRS MLV, Ingelvac PRRS ATP, Fostera PRRS, Prevacent PRRS, and Prime Pac PRRS vaccine viruses as well as an IngelvacMLV/Fostera/Prevacent/XIPC 4-plex real-time RT-PCR for simultaneous detection and differentiation of three most commonly used PRRSV-2 vaccines in the USA. Inclusion of internal positive control XIPC provides an additional quality assurance approach to ensure the accuracy of the PCR results, as described previously [16]. None of the singleplex or the 4-plex vaccine-like PCR assays and the reference screening PCR cross-reacted with any of the 27 non-PRRSV swine viral and bacterial pathogens and PRRSV-1. Notably, the Prime Pac PCR demonstrated reactivity with both the Prime Pac and the PRRSGard vaccine viruses, whereas the PRRSGard PCR only specifically reacted with the PRRSGard vaccine virus without exhibiting cross reactivity. This disparity occurred because the nsp2 region targeted by the Prime Pac PCR is similar between the Prime Pac and PRRSGard vaccine viruses, while the genetic marker in the ORF1b/ORF2 region targeted by the PRRSGard PCR is unique in the PRRSGard vaccine virus and absent in the Prime Pac vaccine virus and other PRRSV strains. Therefore, if a sample is positive with the Prime Pac PCR, it is important to subject the sample to testing via the PRRSGard PCR to confirm the absence of the PRRSGard vaccine virus.

The singleplex and the 4-plex PCR can specifically detect the respective vaccine virus, even if a wild-type virus is co-present in a sample (Table 8). In PRRSV vaccinations and challenge pig studies, it is possible that pigs vaccinated with a PRRSV MLV vaccine still shed a vaccine virus in samples after challenge with a wild-type PRRSV strain. However, PRRSV screening PCR alone cannot differentiate the vaccine virus and wild-type challenge virus. In contrast, vaccine-like PCR assays can help determine whether the post-challenge samples contain the vaccine virus or not. In addition, combining PRRSV screening PCR and vaccine-like PCR and comparing their C_T_ values may make it possible to determine whether the post-challenge samples contain only the challenge virus or have a co-presence of both a challenge virus and a vaccine virus. This approach has been demonstrated to be feasible and successful in a previous PRRSGard vaccination and 1-7-4 wild-type virus challenge study [13]. In addition, the 4-plex PCR can simultaneously detect and distinguish the Ingelvac MLV, Fostera, and Prevacent vaccine viruses if these three vaccine viruses are co-present in a sample (Figure 1). If multiple vaccines are used on a farm or if it is suspected that a vaccine virus not used on the farm is unintentionally introduced, this 4-plex PCR provides a convenient tool to detect and differentiate them without the need to run three separate singleplex PCRs.

ORF5 sequencing via the Sanger method is the most commonly used method to distinguish PRRSV-2 vaccine strains from wild-type strains. However, there is no standard ORF5 nucleotide identity cutoff value to define a vaccine and wild-type viruses. The ORF5 sequence of a vaccine virus used as a reference sequence for comparison in diagnostic laboratories is generally determined from one batch of the vaccine. In the real world, different batches of a vaccine are used in the field, and they do not always have a 100% nucleotide identity to the vaccine reference sequence. In addition, the vaccine virus could evolve during replication in pigs [27,28,29,30]. Based on our experience at the Iowa State University Veterinary Diagnostic Laboratory, a virus detected in a sample occasionally has a 100% ORF5 nucleotide identity to a vaccine reference sequence; however, in most scenarios, the virus detected in a sample from vaccinated pigs does not have a 100% nucleotide identity to the vaccine reference sequence. In a recently published paper [18], we arbitrarily defined any sequence with a ≥98% ORF5 nucleotide identity to a vaccine virus to be vaccine-like, 95–98% to be a vaccine-like suspect, and a <95% ORF5 nucleotide identity to be a wild-type virus. In this study, we used the same criteria to determine the virus identity. When the singleplex and 4-plex vaccine-like PCR results on 45 PRRSV-2 field and laboratory isolates were compared to the ORF5 sequences of those 45 isolates, most of the isolates that had ~95–100% ORF5 nucleotide identities to a vaccine virus were positive with the respective vaccine-like PCR, with consistent results between the ORF5 sequencing and vaccine-like PCRs. However, a few isolates (e.g. #12, #36, and #37) had 96.7–99.5% ORF5 nucleotide identities to a vaccine virus, but were negative with the respective vaccine-like PCR. Further investigations via NGS analysis indicated that these virus isolates were recombinant viruses. One isolate (#14) had an 87.7% ORF5 nucleotide identity to the Fostera vaccine virus, but was positive with the Fostera PCR targeting the nsp2 region; NGS analysis suggested that isolate #14 was also a recombinant virus. The recombinant virus isolate #36 (USA/IA/70388B/2018) was previously described [21]; however, the recombination in isolates #12, #14, and #37 was described for the first time in this study. Recombination between a PRRSV MLV vaccine virus and a field virus, or between two MLV vaccine viruses, or between wild-type viruses has been well documented worldwide [21,31,32,33,34,35,36,37,38,39,40,41]. For some virus isolates with large C_T_ differences between the vaccine-like PCR and the screening PCR (e.g. #26, #27, and #33), NGS analysis confirmed that these isolates were not pure, and they included ≥2 PRRSV sequences. Notably, the conflicting PCR and sequencing results observed for isolates #32 and #13 indicated the need for further improvement in the Ingelvac ATP PCR Assay 2 and Prevacent PCR assay.

Overall, these singleplex and 4-plex vaccine-like PCRs provide an additional tool to help molecularly characterize PRRSV, especially in combination with other methods, such as PRRSV screening PCR and ORF5 sequencing. If a sample is positive with a PRRSV screening PCR but negative with a PRRSV vaccine-like PCR, several interpretations may be considered: (1) the absence of the respective vaccine-like virus, (2) the potential overlook of the respective vaccine-like virus due to a lower sensitivity or mismatches of primers and/or probes of the vaccine-like PCR, or (3) the presence of a recombinant virus whose genomic region targeted with the respective vaccine-like PCR is not derived from that vaccine virus. If a sample is positive with both a PRRSV screening PCR and a vaccine-like PCR, this suggests the presence of the respective vaccine-like or vaccine-derived virus; however, it should be noted that this result does not conclusively rule out the presence of a recombinant virus involving that vaccine virus, nor does it exclude the co-infection with both the respective vaccine-like virus and wild-type virus. However, a comparison of the C_T_ values between the PRRSV screening PCR and the vaccine-like PCR can occasionally reveal the co-presence of both a wild-type and vaccine-like virus within a sample. If there is a conflict between vaccine-like PCR and ORF5 sequencing results, the sample should be flagged for a more thorough characterization via NGS.

## 5. Conclusions

In this study, we developed and validated singleplex vaccine-like PCRs (Ingelvac MLV Assay 2, Ingelvac ATP Assay 2, Fostera assay, Prime PAC assay, and Prevacent assay) and the 4-plex PCR (IngelvacMLV/Fostera/Prevacent/XIPC) for the specific detection of the respective vaccine-like viruses, with comparable performances to the PRRSV screening PCR. The 4-plex PCR allows simultaneous detection and differentiation of three vaccine viruses (Prevacent PRRS, Ingelvac PRRS MLV, and Fostera PRRS), which are currently the most commonly used in the USA, if they are present in the same sample. These vaccine-like PCR assays are also valuable in determining whether the samples collected post challenge in a vaccination/challenge study contain the vaccine virus or not. Overall, PRRSV-2 vaccine-like PCRs provide an additional tool for molecularly detecting and characterizing PRRSV-2.

## Figures and Tables

**Table 1:** Sequences of primers and probes used in this study.

Assay	Primer/Probe Name	Sequence (5′-3′)	Target Region	Amplicon Size	Reference
***Singleplex Ingelvac PRRS MLV PCR***					
Assay 1	IngelvacMLV-RTF	AGCTAACAAATTTGATTGGGCAGT	ORF5	178 bp	[14]
	IngelvacMLV-RTR	ACAGACCGCGTAGATGCTACTTAG	ORF5		
	IngelvacMLV-RTP	FAM/CTTTAGTCA/ZEN/CTGTGTCTACCGCCGGGTTTG/3IABkFQ	ORF5		
Assay 2	IngelvacMLV_F2	TGGCGCCGGCTCTTTT	nsp2	86 bp	This study
	IngelvacMLV_R2	GCTTTTCTTTTTACCGTCCGAAA	nsp2		
	IngelMLV_Prb2ABY	ABY/CGATTTGCCGCCTTCAGATGGC/QSY	nsp2		
***Singleplex Ingelvac PRRS ATP PCR***					
Assay 1	IngelvacATP-RTF	CAACTTGACGCTATGTGAGCTGAAT	ORF5	130 bp	[14]
	IngelvacATP-RTR	ATGGCTAGTGGTGAGTGCACTATAT	ORF5		
	IngelvacATP-RTP	VIC/ATTGGCTGA/ZEN/AAGACAAATTTGATTGGGCATTGGAGACTTT/3IABkFQ	ORF5		
Assay 2	IngelvaATP_F1	CCACCACAGTCGCTCAC	nsp2	94 bp	[15]
	IngelvacATP_R1	CCTGAGATGCACAGCCTTATCA	nsp2		
	IngelvacATP_Prb1	FAM/TCGTGAAATCCAGCAAGCCAA/MGB	nsp2		
***Singleplex Fostera PRRS PCR***					
	FostFor2a-RTF	GACACAAATTTCAGCAGGTGGAA	nsp2	103 bp	This study
	FostRev2a-RTR	TCATATTCAGTCTGTGAGGATGCA	nsp2		
	FostPrb-RTP_VIC	VIC/CAGCAGCGCCGATG/MGB	nsp2		
***Singleplex Prevacent PRRS PCR***					
	Prevacent-RTF	GAAGGTTAAGTCTAGTTGTGGTGATCTG	nsp2	112 bp	This study
	Prevacent-RTR	CACCGGCTCAGGTAAGATCTG	nsp2		
	Prevacent-RTP	FAM/TTTGGCTGGTGGCTC/MGB	nsp2		
***Singleplex Prime Pac PRRS PCR***					
	PrimePAC-RTF2	GCTCAACCGAGAAAAWTGAACAG	nsp2	81 bp	This study
	PrimePAC-RTR2	GCGGCAAATTGGTAAACGTT	nsp2		
	PrimePAC-RTP2	FAM/CCAGCACACAGGCGT/MGB	nsp2		
***Singleplex PRRSGard PCR***					
	PRRSGard-F	CGGGCCCTGTCATTGAAC	ORF1b/2	65 bp	[13]
	PRRSGard-R	CATTGGCGCGCTATTTAAATTA	ORF1b/2		
	PRRSGard-Prb	FAM/CTTTAGGCCTGAATTGATTAA/MGB	ORF1b/2		
***IngelvacMLV/Fostera/Prevacent/XIPC 4-plex PCR***					
	IngelvacMLV_F2	TGGCGCCGGCTCTTTT	nsp2	86 bp	This study
	IngelvacMLV_R2	GCTTTTCTTTTTACCGTCCGAAA	nsp2		
	IngelMLV_Prb2ABY	**ABY**/CGATTTGCCGCCTTCAGATGGC/**QSY**	nsp2		
	FostFor2a-RTF	GACACAAATTTCAGCAGGTGGAA	nsp2	103 bp	This study
	FostRev2a-RTR	TCATATTCAGTCTGTGAGGATGCA	nsp2		
	FostPrb-RTP_VIC	**VIC**/CAGCAGCGCCGATG/**MGB**	nsp2		
	Prevacent-RTF	GAAGGTTAAGTCTAGTTGTGGTGATCTG	nsp2	112 bp	This study
	Prevacent-RTR	CACCGGCTCAGGTAAGATCTG	nsp2		
	Prevacent-RTP	**FAM**/TTTGGCTGGTGGCTC/**MGB**	nsp2		

Note: The singleplex PCR assays used to develop the 4-plex PCR are highlighted in green.

**Table 2:** Analytical specificity of various singleplex vaccine-like PCRs and 4-plex PCR.

Pathogens	Singleplex PCR C_T_								IngelvacMLV /Fostera /Prevacent/XIPC 4-Plex PCR C_T_	Reference PRRSV Screening PCR C_T_	
	Ingelvac MLV Assay 1	Ingelvac MLV Assay 2	Ingelvac ATP Assay 1	Ingelvac ATP Assay 2	Fostera	Prevacent	Prime Pac	PRRS Gard		PRRSV-2	PRRSV-1
Ingelvac PRRS MLV vaccine	22.7	20.5	≥40	≥40	≥40	≥40	≥40	≥40	21.2/≥40/≥40/22.5	20.4	≥40
Ingelvac PRRS ATP vaccine	≥40	≥40	21.5	22.3	≥40	≥40	≥40	≥40	≥40/≥40/≥40/22.3	19.4	≥40
Fostera PRRS vaccine	≥40	≥40	36.9	≥40	20.4	≥40	≥40	≥40	≥40/21.6/≥40/22.4	18.2	≥40
Prevacent PRRS vaccine	32.1	≥40	≥40	≥40	≥40	15.7	≥40	≥40	≥40/≥40/16.9/22.4	16.5	≥40
Prime Pac PRRS vaccine	≥40	≥40	≥40	≥40	≥40	≥40	24.1	≥40	≥40/≥40/≥40/22.3	22.7	≥40
PRRSGard vaccine	≥40	≥40	≥40	≥40	≥40	≥40	15.2	15.1	≥40/≥40/≥40/22.4	13.6	≥40
PRRSV-1_Lelystad	≥40	≥40	≥40	≥40	≥40	≥40	≥40	≥40	≥40/≥40/≥40/22.5	≥40	12.6
Non-PRRSV swine viruses †	≥40	≥40	≥40	≥40	≥40	≥40	≥40	≥40	≥40/≥40/≥40/22.3–22.5	≥40	≥40
Bacteria ‡	≥40	≥40	≥40	≥40	≥40	≥40	≥40	≥40	≥40/≥40/≥40/22.3–22.5	≥40	≥40

Red-colored font: vaccine-like PCR assays had cross-reactivity to some PRRSV strains. † Non-PRRSV swine viruses for evaluating assay specificity include influenza A virus, porcine circovirus 2, porcine circovirus 3, porcine parainfluenza virus 1, porcine respiratory coronavirus, porcine hemagglutinating encephalomyelitis virus, pseudorabies virus, porcine epidemic diarrhea virus, transmissible gastroenteritis virus Purdue strain and Miller strain, porcine deltacoronavirus, porcine rotavirus A, B, C, and Seneca Valley virus. ‡ Bacterial pathogens for evaluating assay specificity include *Mycoplasma hyopneumoniae*, *Mycoplasma hyorhinis*, *Mycoplasma hyosynoviae*, *Pasteurella multocida*, *Streptococcus suis*, *Glaesserella (Haemophilus) parasuis*, *Bordetella bronchiseptica*, *Actinobacillus pleuropneumonia*, *Actinobacillus suis*, *Trueperella pyogenes*, *E. coli*, *Salmonella typhimurium*, *Clostridium difficile*, *Clostridium perfringens*, and *Brachyspira hyodysenteriae*.

**Table 3:** Analytical sensitivity of the singleplex and 4-plex vaccine-like PCR assays using serial dilutions of MLV vaccine viruses.

PCR	Vaccine Virus and Replicate	Serial Dilutions of MLV Vaccine Virus and PCR C_T_ Value						
		10^−1^	10^−2^	10^−3^	10^−4^	10^−5^	10^−6^	10^−7^
Reference PRRSV screening PCR	Ingelvac MLV Rep 1	22.7	26.5	30.4	34.0	35.6	≥40	≥40
	Ingelvac MLV Rep 2	22.5	26.4	30.5	33.9	35.6	≥40	≥40
	Ingelvac MLV Rep 3	22.3	26.6	30.4	33.9	35.4	≥40	≥40
Ingelvac MLV singleplex PCR Assay 2 (ABY)	Ingelvac MLV Rep 1	24.3	27.9	31.1	34.4	≥40	≥40	≥40
	Ingelvac MLV Rep 2	24.4	28.0	31.5	35.5	36.5	≥40	≥40
	Ingelvac MLV Rep 3	24.3	27.8	31.3	34.6	36.2	≥40	≥40
IngelvacMLV/Fostera/Prevacent/XIPC 4-plex PCR	Ingelvac MLV Rep 1	24.8	28.7	33.3	36.4	≥40	≥40	≥40
	Ingelvac MLV Rep 2	24.4	28.9	32.7	34.9	≥40	≥40	≥40
	Ingelvac MLV Rep 3	24.5	28.7	32.6	34.7	≥40	≥40	≥40
Reference PRRSV screening PCR	IngelvacATP Rep 1	22.9	25.7	29.0	33.0	36.2	≥40	≥40
	IngelvacATP Rep 2	22.6	25.6	29.0	32.7	36.6	≥40	≥40
	IngelvacATP Rep 3	22.8	25.7	29.2	32.7	36.5	≥40	≥40
Ingelvac ATP singleplex PCR Assay 2 (FAM)	Ingelvac ATP Rep 1	23.0	26.6	30.2	33.8	≥40	≥40	≥40
	Ingelvac ATP Rep 2	23.0	26.6	30.0	33.7	36.9	≥40	≥40
	Ingelvac ATP Rep 3	23.0	26.7	30.1	33.9	≥40	≥40	≥40
Reference PRRSV screening PCR	Fostera Rep 1	20.7	23.2	27.4	31.2	36.0	≥40	≥40
	Fostera Rep 2	20.6	23.3	27.4	31.3	36.2	≥40	≥40
	Fostera Rep 3	20.7	23.3	27.7	31.4	36.1	≥40	≥40
Fostera singleplex PCR (VIC)	Fostera Rep 1	23.1	26.4	29.8	32.7	35.6	39.3	≥40
	Fostera Rep 2	23.2	26.5	29.9	32.8	35.7	37.9	≥40
	Fostera Rep 3	23.2	26.6	30.0	32.8	35.4	39.3	≥40
IngelvacMLV/Fostera/Prevacent/XIPC 4-plex PCR	Fostera Rep 1	21.1	24.3	29.9	33.0	36.5	≥40	≥40
	Fostera Rep 2	21.1	24.4	29.0	33.3	36.3	≥40	≥40
	Fostera Rep 3	21.1	24.4	29.1	33.2	36.6	≥40	≥40
Reference PRRSV screening PCR	Prevacent Rep 1	19.4	23.2	28.7	30.2	33.1	36.5	≥40
	Prevacent Rep 2	19.3	23.1	28.6	30.2	33.0	36.0	≥40
	Prevacent Rep 3	19.3	23.1	28.6	30.2	34.0	36.8	≥40
Prevacent singleplex PCR (FAM)	Prevacent Rep 1	19.8	22.9	26.4	30.1	34.0	≥40	≥40
	Prevacent Rep 2	20.0	23.0	26.4	30.1	33.5	≥40	≥40
	Prevacent Rep 3	19.9	22.9	26.4	30.0	33.6	≥40	≥40
IngelvacMLV/Fostera/Prevacent/XIPC 4-plex PCR	Prevacent Rep 1	20.3	23.7	26.5	30.8	34.0	≥40	≥40
	Prevacent Rep 2	20.2	23.7	26.6	30.9	35.6	≥40	≥40
	Prevacent Rep 3	20.2	23.9	26.4	30.7	35.9	≥40	≥40
Reference PRRSV screening PCR	Prime Pac Rep 1	25.0	26.2	29.7	33.4	38.8	≥40	≥40
	Prime Pac Rep 2	24.9	26.5	29.7	33.4	38.8	≥40	≥40
	Prime Pac Rep 3	24.9	26.2	29.7	33.4	38.8	≥40	≥40
Prime Pac singleplex PCR (FAM)	Prime Pac Rep 1	25.0	28.1	31.6	36.0	≥40	≥40	≥40
	Prime Pac Rep 2	25.0	28.1	31.8	34.2	37.0	≥40	≥40
	Prime Pac Rep 3	25.0	28.2	31.8	35.0	≥40	≥40	≥40

Red-colored font: virus dilution that was the endpoint detection using the respective vaccine-like PCR and the reference screening PCR.

**Table 4:** Limit of detection of the singleplex and 4-plex vaccine-like PCRs using serial dilutions of in vitro transcribed RNA.

Genomic Copies per Reaction	Singleplex Vaccine-like PCR		IngelvacMLV/Fostera/Prevacent/XIPC 4-Plex PCR	
***Ingelvac MLV partial nsp2 IVT RNA***	% (No. of Pos for Ingelvac MLV)	Mean C_T_ range	% (No. of Pos for Ingelvac MLV)	Mean C_T_ (range)
5 × 10^4^	100% (3/3)	29.7 (28.6–30.9)	100% (3/3)	29.9 (29.6–30.6)
5 × 10^3^	100% (3/3)	33.5 (32.3–35.0)	100% (3/3)	34.1 (33.1–35.5)
5 × 10^2^	100% (3/3)	35.9 (34.9–36.9)	100% (3/3)	36.7 (35.5–37.0)
5 × 10^1^	100% (20/20)	37.8 (36.1–39.0)	100% (20/20)	38.3 (37.4–39.0)
25	80% (16/20)	38.4 (36.7–40)	60% (12/20)	39.1 (38.6–40)
12.5	35% (7/20)	39.4 (37.6–40)	20% (4/20)	39.8 (39.8–40)
6.25	10% (2/20)	39.9 (38.8–40)	0% (0/20)	40 (40)
***Ingelvac ATP partial nsp2 IVT RNA***	% (No. of Pos for Ingelvac ATP)	Mean C_T_ range		
5 × 10^4^	100% (3/3)	23.8 (23.7–24.0)		
5 × 10^3^	100% (3/3)	27.1 (27.0–27.2)		
5 × 10^2^	100% (3/3)	30.7 (30.5–30.9)		
5 × 10^1^	100% (20/20)	35.2 (32.8–38.3)		
25	85% (17/20)	36.3 (33.9–40)		
12.5	65% (13/20)	37.8 (35.0–40)		
6.25	40% (8/20)	38.7 (34.4–40)		
***Fostera partial nsp2 IVT RNA***	% (No. of Pos for Fostera)	Mean C_T_ range	% (No. of Pos for Fostera)	Mean C_T_ (range)
5 × 10^4^	100% (3/3)	25.6 (25.3–25.8)	100% (3/3)	25.8 (25.3–26.1)
5 × 10^3^	100% (3/3)	28.7 (28.8–28.9)	100% (3/3)	29.0 (28.9–29.10)
5 × 10^2^	100% (3/3)	32.1 (32.0–32.2)	100% (3/3)	32.2 (31.2–32.8)
5 × 10^1^	95% (19/20)	36.8 (35.1–40)	95% (19/20)	37.1 (36.9–40)
25	80% (16/20)	38.5 (36.4–40)	65% (13/20)	38.7 (38.2–40)
12.5	55% (11/20)	39.1 (37.3–40)	40% (8/20)	39.9 (39.8–40)
6.25	25% (5/20)	39.6 (35.8–40)	0% (0/20)	40 (40)
***Prevacent partial nsp2 IVT RNA***	% (No. of Pos for Prevacent)	Mean C_T_ range	% (No. of Pos for Prevacent)	Mean C_T_ (range)
5 × 10^4^	100% (3/3)	26.5 (26.1–27.01)	100% (3/3)	26.9 (26.5–27.6)
5 × 10^3^	100% (3/3)	30.1 (29.8–30.5)	100% (3/3)	30.1 (29.8–30.5)
5 × 10^2^	100% (3/3)	33.9 (33.8–34.1)	100% (3/3)	34.0 (33.6–34.4)
5 × 10^1^	100% (20/20)	35.0 (33.8–36.8)	100% (20/20)	35.7 (35.1–36.9)
25	100% (20/20)	36.2 (35.1–38.4)	100% (20/20)	37.9 (37.8–38.0)
12.5	80% (16/20)	36.8 (35.1–40)	75% (15/20)	38.8 (38.5–39.0)
6.25	60% (12/20)	37.9 (35.6–40)	50% (10/20)	38.9 (38.8–40)
***Prime Pac partial nsp2 IVT RNA***	% (No. of Pos for Prime Pac)	Mean C_T_ range		
5 × 10^4^	100% (3/3)	25.9 (25.8–26.2)		
5 × 10^3^	100% (3/3)	29.3 (29.2–29.5)		
5 × 10^2^	100% (3/3)	32.6 (32.2–33.2)		
5 × 10^1^	95% (19/20)	36.6 (34.4–40)		
25	60% (12/20)	38.1 (35.7–40)		
12.5	20% (4/20)	39.4 (37.0–40)		
6.25	25% (5/20)	39.2 (35.5–40)		

Light green color: the genomic copies/reactions corresponding to the limit of detection of each PCR assay.

**Table 5:** Repeatability of the singleplex and 4-plex vaccine-like PCRs using serial dilutions of in vitro transcribed RNA.

Target	IVT RNA (Genomic Copies/Reactions)	Respective Singleplex Vaccine-like PCR				IngelvacMLV/Fostera/Prevacent/XIPC 4-Plex PCR			
		Intra-Assay (3 Replicates for Each Dilution)		Inter-Assay (3 Plates for Each Dilution)		Intra-assay (3 Replicates for Each Dilution)		Inter-Assay (3 Plates for Each Dilution)	
		Mean C_T_ value ± SD	CV, %	Mean C_T_ value ± SD	CV, %	Mean C_T_ value ± SD	CV, %	Mean C_T_ value ± SD	CV, %
Ingelvac PRRS MLV nsp2	5 × 10^4^	29.70 ± 0.49	1.65	29.72 ± 0.03	0.09	29.76 ± 0.74	2.48	29.95 ± 0.40	1.33
	5 × 10^3^	33.35 ± 0.37	1.10	33.20 ± 0.34	1.02	34.14 ± 0.35	1.02	33.95 ± 0.29	0.85
	5 × 10^2^	35.91 ± 0.48	1.33	35.52 ± 0.33	0.93	36.75 ± 0.49	1.35	36.16 ± 0.70	1.95
	5 × 10^1^	37.85 ± 0.31	0.82	37.68 ± 0.38	1.01	38.30 ± 0.18	0.47	38.33 ± 0.41	1.08
		Average CV, %	1.22	Average CV, %	0.76	Average CV, %	1.33	Average CV, %	1.30
Ingelvac PRRS ATP nsp2	5 × 10^4^	23.21 ± 0.32	1.38	23.51 ± 0.51	2.17				
	5 × 10^3^	26.96 ± 0.35	1.31	27.07 ± 0.23	0.86				
	5 × 10^2^	30.57 ± 0.50	1.64	31.17 ± 0.66	2.10				
	5 × 10^1^	36.28 ± 0.52	1.43	36.25 ± 0.46	1.27				
		Average CV, %	1.44	Average CV, %	1.60				
Fostera PRRS nsp2	5 × 10^4^	25.31 ± 0.17	0.68	25.76 ± 0.20	0.78	25.82 ± 0.33	1.29	25.86 ± 0.25	0.97
	5 × 10^3^	28.44 ± 0.18	0.64	28.47 ± 0.34	1.18	28.63 ± 0.41	1.45	28.66 ± 0.31	1.07
	5 × 10^2^	32.22 ± 0.04	0.12	32.60 ± 0.31	0.95	32.57 ± 0.28	0.87	32.74 ± 0.39	1.18
	5 × 10^1^	37.26 ± 0.40	1.07	37.50 ± 0.34	0.92	37.91 ± 0.22	0.58	38.03 ± 0.46	1.22
		Average CV, %	0.63	Average CV, %	0.96	Average CV, %	1.05	Average CV, %	1.11
Prevacent PRRS nsp2	5 × 10^4^	26.52 ± 0.13	0.49	26.39 ± 0.12	0.45	27.18 ± 0.27	1.00	27.49 ± 0.41	1.50
	5 × 10^3^	28.94 ± 0.45	1.57	28.57 ± 0.09	0.33	29.57 ± 0.12	0.42	29.66 ± 0.05	0.18
	5 × 10^2^	31.67 ± 0.30	0.94	31.79 ± 0.24	0.74	32.98 ± 0.48	1.45	33.95 ± 0.19	0.55
	5 × 10^1^	35.02 ± 0.27	0.78	35.09 ± 0.52	1.48	35.95 ± 0.82	2.27	36.03 ± 0.18	0.51
		Average CV, %	0.95	Average CV, %	0.75	Average CV, %	1.29	Average CV, %	0.68
Prime Pac PRRS nsp2	5 × 10^4^	25.51 ± 0.07	0.26	25.71 ± 0.10	0.38				
	5 × 10^3^	29.64 ± 0.11	0.38	29.52 ± 0.45	1.52				
	5 × 10^2^	32.80 ± 0.23	0.70	32.55 ± 0.36	1.12				
	5 × 10^1^	36.89 ± 0.63	1.70	36.72 ± 0.32	0.88				
		Average CV, %	0.76	Average CV, %	0.98				

**Table 6:** Diagnostic performance of the singleplex and 4-plex PCRs in comparison with the reference PRRSV screening PCR on clinical samples *.

		Reference PRRSV Screening PCR		
		Pos	Neg	Total
Ingelvac MLV singleplex PCR Assay 2	Pos	65	0	65
	Neg	1	114	115
	Total	66	114	180 ^a^
Sensitivity 98.48%; specificity 100%; agreement 99.44%				
Ingelvac ATP singleplex PCR Assay 2	Pos	63	0	63
	Neg	1	107	108
	Total	64	107	171 ^b^
Sensitivity 98.44%; specificity 100%; agreement 99.42%				
Fostera singleplex PCR	Pos	75	0	75
	Neg	4	101	105
	Total	79	101	180 ^c^
Sensitivity 94.94%; specificity 100%; agreement 97.78%				
Prevacent singleplex PCR	Pos	73	0	73
	Neg	0	107	107
	Total	73	107	180 ^d^
Sensitivity 100%; specificity 100%; agreement 100%				
Prime Pac singleplex PCR	Pos	71	0	71
	Neg	4	105	109
	Total	75	105	180 ^e^
Sensitivity 94.67%; specificity 100%; agreement 97.78%				
4-plex PCR—**Ingelvac MLV**	Pos	65	0	65
	Neg	1	114	115
	Total	66	114	180 ^a^
Sensitivity 98.48%; specificity 100%; agreement 99.44%				
4-plex PCR—**Fostera**	Pos	75	0	75
	Neg	4	101	105
	Total	79	101	180 ^c^
Sensitivity 94.94%; specificity 100%; agreement 97.78%				
4-plex PCR—**Prevacent**	Pos	73	0	73
	Neg	0	107	107
	Total	73	107	180 ^d^
Sensitivity 100%; specificity 100%; agreement 100%				

* For all PCRs, Ct < 37 was considered as positive. ^a^ 180 serum samples collected at 0, 2, 4, 7, 10, 14, 21, 28, and 35 days post vaccination (DPV) from 10 mock-vaccinated pigs and 10 pigs vaccinated with Ingelvac MLV; ^b^ 171 serum samples collected from 10 mock-vaccinated pigs and 9 pigs vaccinated with Ingelvac ATP; ^c^ 180 serum samples collected from 10 mock-vaccinated pigs and 10 Fostera-vaccinated pigs; ^d^ 180 serum samples collected from 10 mock-vaccinated pigs and 10 Prevacent-vaccinated pigs; ^e^ 180 serum samples collected from 10 mock-vaccinated pigs and 10 pigs vaccinated with Prime Pac.

**Table 7:** Discrepancies in clinical samples between the PRRSV-2 singleplex vaccine-like PCRs, 4-plex PCR, and the reference PRRSV screening PCR *.

Sample ID	Specimen	Reference PRRSV Screening PCR C_T_	Singleplex Vaccine-like PCR C_T_ Value					4-Plex PCR C_T_ Value		
			Ingelvac MLV	Ingelvac ATP	Fostera	Prevacent	Prime Pac	Ingelvac MLV	Fostera	Prevacent
Sample_#1	Serum	36.4	≥37	ND	ND	ND	ND	≥37	≥37	≥37
Sample_#2	Serum	34.9	ND	≥37	ND	ND	ND	ND	ND	ND
Sample_#3	Serum	35.8	ND	ND	≥37	ND	ND	≥37	≥37	≥37
Sample_#4	Serum	36.2	ND	ND	≥37	ND	ND	≥37	≥37	≥37
Sample_#5	Serum	35.7	ND	ND	≥37	ND	ND	≥37	≥37	≥37
Sample_#6	Serum	36.0	ND	ND	≥37	ND	ND	≥37	≥37	≥37
Sample_#7	Serum	36.9	ND	ND	ND	ND	≥37	ND	ND	ND
Sample_#8	Serum	36.2	ND	ND	ND	ND	≥37	ND	ND	ND
Sample_#9	Serum	35.0	ND	ND	ND	ND	≥37	ND	ND	ND
Sample_#10	Serum	35.6	ND	ND	ND	ND	≥37	ND	ND	ND

* For all PCRs, C_T_ < 37 was considered as positive. ND: not done. Sample_#1 was from the Ingelvac PRRS MLV vaccination group; Sample_#2 was from the Ingelvac PRRS ATP vaccination group; Sample_#3–#6 were from the Fostera PRRS vaccination group; and Sample_#7–#10 were from the Prime Pac PRRS vaccination group.

**Table 8:** IngelvacMLV/Fostera/Prevacent/XIPC 4-plex and the reference PRRSV screening PCR on manual mixtures of vaccine viruses and a wild-type PRRSV isolate at different ratios.

Manual Mix of Viruses	Concentration Ratio	Ingelvac MLV(ABY)/Fostera(VIC)/Prevacent(FAM)/XIPC(Cy5) 4-Plex PCR C_T_	Reference PRRSV Screening PCR ^†^ PRRSV-2 C_T_
Wild-type PRRSV isolate stock *	N/A	≥40/≥40/≥40/29.2	16.1
Ingelvac PRRS MLV vaccine virus	N/A	**21.6**/≥40/≥40/29.4	18.1
Fostera PRRS vaccine virus	N/A	≥40/**20.9**/≥40/29.8	16.6
Prevacent PRRS vaccine virus	N/A	≥40/≥40/**15.0**/29.2	15.1
Mix_1 (Ingelvac MLV + wild-type)	1:1	**22.4**/≥40/≥40/29.5	16.8
Mix_2 (Ingelvac MLV + wild-type)	10^−1^:1	**26.3**/≥40/≥40/29.7	17.2
Mix_3 (Ingelvac MLV + wild-type)	10^−2^:1	**29.7**/≥40/≥40/30.1	17.6
Mix_4 (Ingelvac MLV + wild-type)	10^−3^:1	**32.6**/≥40/≥40/29.9	17.7
Mix_5 (Ingelvac MLV + wild-type)	10^−4^:1	**36.3**/≥40/≥40/30.2	17.9
Mix_6 (Ingelvac MLV + wild-type)	10^−5^:1	≥40/≥40/≥40/29.5	17.6
Mix_7 (Ingelvac MLV + wild-type)	10^−6^:1	≥40/≥40/≥40/29.8	17.6
Mix_8 (Ingelvac MLV + wild-type)	10^−7^:1	≥40/≥40/≥40/29.8	17.8
Mix_9 (Fostera + wild-type)	1:1	≥40/**21.8**/≥40/30.0	16.3
Mix_10 (Fostera + wild-type)	10^−1^:1	≥40/**25.2**/≥40/30.6	17.1
Mix_11 (Fostera + wild-type)	10^−2^:1	≥40/**27.9**/≥40/29.3	17.2
Mix_12 (Fostera + wild-type)	10^−3^:1	≥40/**31.6**/≥40/29.6	17.3
Mix_13 (Fostera + wild-type)	10^−4^:1	≥40/**33.8**/≥40/30.0	18.0
Mix_14 (Fostera + wild-type)	10^−5^:1	≥40/≥40/≥40/29.5	17.6
Mix_15 (Fostera + wild-type)	10^−6^:1	≥40/≥40/≥40/30.0	17.6
Mix_16 (Fostera + wild-type)	10^−7^:1	≥40/≥40/≥40/29.9	17.5
Mix_17 (Prevacent + wild-type)	1:1	≥40/≥40/**16.0**/30.8	15.0
Mix_18 (Prevacent + wild-type)	10^−1^:1	≥40/≥40/**19.8**/29.3	16.8
Mix_19 (Prevacent + wild-type)	10^−2^:1	≥40/≥40/**22.9**/30.0	17.5
Mix_20 (Prevacent + wild-type)	10^−3^:1	≥40/≥40/**26.5**/29.3	17.3
Mix_21 (Prevacent + wild-type)	10^−4^:1	≥40/≥40/**29.7**/30.2	17.3
Mix_22 (Prevacent + wild-type)	10^−5^:1	≥40/≥40/**34.4**/30.1	18.0
Mix_23 (Prevacent + wild-type)	10^−6^:1	≥40/≥40/**37.0**/30.6	18.2
Mix_24 (Prevacent + wild-type)	10^−7^:1	≥40/≥40/≥40/29.6	17.4

* A field PRRSV-2 isolate ISU14-65239 with RFLP pattern of 1-7-4 was used as a wild-type strain; ^†^ the reference PRRSV screening PCR: VetMAX PRRSV NA&EU Reagent 2.0 (Thermo Fisher Scientific).

**Table 9:** Information of lineage and RFLP typing of various PRRSV-2 isolates and their ORF5 nucleotide identity in relation to six PRRSV-2 MLV vaccine viruses.

Series No.	PRRSV-2 Isolates	ORF5-Based Lineage	ORF5-Based RFLP Typing	ORF5 nt Identity (%) to Ingelvac MLV	ORF5 nt Identity (%) to Ingelvac ATP	ORF5 nt Identity (%) to Fostera	ORF5 nt Identity (%) to Prevacent	ORF5 nt Identity (%) to Prime Pac	ORF5 nt Identity (%) to PRRSGard
#1	USA/IA/79622-4/2019	L1A	1-6-2	88.9	87.6	88.9	89.4	88.6	88.9
#2	USA/IN/65239GA/2014	L1A	1-7-4	87.7	86.4	86.4	88.9	87.9	90.4
#3	USA/IA/14671GA/2016	L1A	1-4-4	88.6	88.1	88.1	89.9	88.7	90.7
#4	USA/IA/23143-7/2017	L1B	1-18-2	84.4	85.1	84.4	84.9	83.9	86.9
#5	Mexico/49783GB/2019	L1B	1-26-2	84.9	84.9	84.9	85.4	84.7	86.4
#6	USA/IN/12110GA/2019	L1C	1-3-4	85.7	85.4	86.1	87.9	85.9	86.1
#7	USA/IA/79039-3/2019	L1C	1-18-4	83.1	83.7	84.4	86.9	83.7	86.9
#8	USA/NE/05828-3/2020	L1C.1	1-4-4	85.1	84.4	84.9	87.2	85.4	88.6
#9	USA/MN/01775GA/2021	L1C.5	1-4-4	86.4	85.9	86.7	89.4	86.9	88.7
#10 *	USA/IA/104589-1/2021	L1D	1-8-4	86.6	85.4	86.2	99.3	86.7	89.7
#11 *	USA/IA/90715-5/2021	L1D	1-12-4	86.1	84.1	85.4	98.2	86.2	88.1
#12 *	USA/IN/04584GF/2022	L1D	1-8-4	86.1	84.2	85.4	96.7	86.7	87.6
#13 *	USA/IL/01810A/2012	L1D	1-12-4	87.2	85.4	86.7	94.5	87.9	88.4
#14 *	USA/AZ/74959GA/2020	L1E	1-3-2	86.2	84.9	87.7	85.6	86.1	84.6
#15	USA/KY/71761/2015	L1E	1-22-2	86.2	86.1	86.7	86.1	86.1	86.4
#16 *	USA/IL/17142GA/2022	L1F	1-8-4	86.2	86.2	86.7	89.7	86.7	100.0
#17 *	USA/MN184	L1F	1-8-4	86.6	86.6	87.1	90.0	87.1	99.7
#18	USA/MO/56050-3/2018	L1F	1-8-4	86.1	85.4	85.1	90.4	85.9	96.0
#19	USA/IA/36983-3/2014	L1F	1-8-4	85.6	85.6	86.1	88.4	85.2	95.5
#20	USA/IA/19170-2/2014	L1F	1-12-4	85.3	85.2	85.2	88.3	85.3	94.5
#21	USA/IA/95000GA/2019	L1H	1-8-4	85.6	85.7	86.6	89.4	87.1	90.2
#22	USA/81793-6/2019	L1H	1-4-4	84.7	85.6	85.4	88.4	85.9	88.4
#23 *	USA/IL/22102-2/2018	L5A	2-5-2	99.5	90.2	91.4	86.7	91.7	86.2
#24	USA/KS/53881/2020	L5A	2-5-2	98.8	90.0	92.0	86.6	91.9	86.4
#25	USA/IN/17168-1/2020	L5A	2-1-2	98.0	89.6	91.0	86.4	91.4	86.1
#26 *	USA/KY/47082-2/2020	L5A	2-6-2	97.7	89.4	91.0	86.2	91.2	86.1
#27 *	USA/PA/60596-1/2020	L5A	2-1-2	96.5	88.4	89.9	85.1	90.4	84.6
#28 *	USA/TN/45339-3/2021	L5A	2-5-4	94.2	88.6	89.7	84.4	89.4	84.6
#29	USA/69077-1/2019	L8A	1-4-2	90.2	100.0	93.7	85.4	89.7	86.2
#30	USA/IL/101561-52/2022	L8A	1-4-2	90.9	99.0	94.5	86.1	90.7	86.9
#31	USA/OK/34563GB/2021	L8A	1-4-2	90.4	98.2	93.0	85.6	89.9	86.6
#32 *	USA/IN/57008/2013	L8A	1-7-4	90.4	97.7	93.0	85.6	90.9	86.2
#33 *	USA/IN/26125/2012	L8A	1-2-2	89.6	96.8	92.0	84.6	88.4	85.1
#34	USA/SDSU73	L8B	1-4-4	89.9	92.0	94.3	85.9	91.8	87.6
#35	USA/IA/24815/2018	L8C	1-3-2	91.5	93.5	99.7	86.6	93.0	86.7
#36 *	USA/IA/70388B/2018	L8C	1-3-2	91.2	93.4	99.5	86.8	92.9	86.6
#37 *	USA/IA/93743-1/2020	L8C	1-4-1	90.9	93.5	99.0	86.2	92.5	86.7
#38	USA/UT/88120-4/2019	L8C	1-4-2	91.5	92.9	98.0	87.4	93.2	86.9
#39	USA/UT/76106-4/2021	L8C	1-4-1	90.9	92.5	97.7	86.9	92.5	86.2
#40	USA/UT/64317GA/2021	L8C	1-4-2	90.9	92.2	97.2	86.7	92.4	86.7
#41	USA/UT/18316GA/2021	L8C	1-1-1	89.9	91.5	96.2	85.9	91.5	85.4
#42 *	USA/UT/34926-1/2021	L8C	1-3-1	89.4	90.7	95.4	85.4	91.0	85.2
#43	Mexico/22470-1/2019	L8D	1-7-3	84.1	85.9	86.9	83.3	84.7	83.6
#44	USA/16572/2017	L9	1-13-2	88.6	87.7	90.9	84.6	90.5	84.2
#45	USA/KY/71236GA/2020	L9A	1-2-4	86.6	88.2	90.5	85.9	89.1	83.9

* Isolates whose whole genome sequences were included for analysis in this study. Red-colored font: samples with discrepant results between ORF5 sequencing and vaccine-like PCR. The samples with >94% ORF5 nucleotide identity to a vaccine virus are shown by blue ground color.

**Table 10:** Various PRRSV-2 isolates tested with singleplex vaccine-like PCRs, 4-plex PCR, and the reference PRRSV screening PCR.

Series No.	PRRSV-2 Isolates	Singleplex PCR C_T_						IngelvacMLV/Fostera/Prevacent/XIPC 4-Plex PCR C_T_	Reference Screening PCR C_T_ (PRRSV-2)
		Ingelvac MLV Assay 2	Ingelvac ATP Assay 2	Fostera	Preva cent	Prime Pac	PRRS Gard		
#1	USA/IA/79622-4/2019	≥40	≥40	≥40	≥40	≥40	≥40	≥40/≥40/≥40/22.5	17.1
#2	USA/IN/65239GA/2014	≥40	≥40	≥40	≥40	≥40	≥40	≥40/≥40/≥40/22.4	14.2
#3	USA/IA/14671GA/2016	≥40	≥40	≥40	≥40	≥40	≥40	≥40/≥40/≥40/22.3	15.8
#4	USA/IA/23143-7/2017	≥40	≥40	≥40	≥40	≥40	≥40	≥40/≥40/≥40/22.4	12.1
#5	Mexico/49783GB/2019	≥40	≥40	≥40	≥40	≥40	≥40	≥40/≥40/≥40/22.4	23.1
#6	USA/IN/12110GA/2019	≥40	≥40	≥40	≥40	≥40	≥40	≥40/≥40/≥40/22.4	18.5
#7	USA/IA/79039-3/2019	≥40	≥40	≥40	≥40	≥40	≥40	≥40/≥40/≥40/22.3	14.0
#8	USA/NE/05828-3/2020	≥40	≥40	≥40	≥40	≥40	≥40	≥40/≥40/≥40/22.4	14.6
#9	USA/MN/01775GA/2021	≥40	≥40	≥40	≥40	≥40	≥40	≥40/≥40/≥40/22.4	13.7
#10	USA/IA/104589-1/2021	≥40	≥40	≥40	10.8	≥40	≥40	≥40/≥40/11.1/22.4	11.9
#11	USA/IA/90715-5/2021	≥40	≥40	≥40	13.5	≥40	≥40	≥40/≥40/13.5/22.4	12.4
#12	USA/IN/04584GF/2022	≥40	≥40	≥40	≥40	≥40	≥40	≥40/≥40/≥40/22.3	13.4
#13	USA/IL/01810A/2012	≥40	≥40	≥40	24.3	≥40	≥40	≥40/≥40/24.9/22.5	20.1
#14	USA/AZ/74959GA/2020	≥40	≥40	20.1	≥40	≥40	≥40	≥40/20.6/≥40/22.4	20.6
#15	USA/KY/71761/2015	≥40	≥40	≥40	≥40	≥40	≥40	≥40/≥40/≥40/22.5	15.0
#16	USA/IL/17142GA/2022	≥40	≥40	≥40	≥40	≥40	≥40	≥40/≥40/≥40/22.4	14.0
#17	USA/MN184	≥40	≥40	≥40	≥40	≥40	≥40	≥40/≥40/≥40/22.5	13.1
#18	USA/MO/56050-3/2018	≥40	≥40	≥40	≥40	≥40	≥40	≥40/≥40/≥40/22.3	17.5
#19	USA/IA/36983-3/2014	≥40	≥40	≥40	≥40	≥40	≥40	≥40/≥40/≥40/22.4	15.1
#20	USA/IA/19170-2/2014	≥40	≥40	≥40	≥40	≥40	≥40	≥40/≥40/≥40/22.4	27.2
#21	USA/IA/95000GA/2019	≥40	≥40	≥40	≥40	≥40	≥40	≥40/≥40/≥40/22.4	21.4
#22	USA/81793-6/2019	≥40	≥40	≥40	≥40	≥40	≥40	≥40/≥40/≥40/22.4	20.6
#23	USA/IL/22102-2/2018	13.7	≥40	≥40	≥40	≥40	≥40	15.7/≥40/≥40/22.3	13.0
#24	USA/KS/53881/2020	16.5	≥40	≥40	≥40	≥40	≥40	20.1/≥40/≥40/22.3	13.8
#25	USA/IN/17168-1/2020	18.2	≥40	≥40	≥40	≥40	≥40	19.6/≥40/≥40/22.5	17.1
#26	USA/KY/47082-2/2020	21.2	≥40	≥40	≥40	≥40	≥40	21.5/≥40/≥40/22.3	10.3
#27	USA/PA/60596-1/2020	25.7	≥40	≥40	≥40	≥40	≥40	27.0/≥40/≥40/22.4	15.8
#28	USA/TN/45339-3/2021	13.7	≥40	≥40	≥40	≥40	≥40	13.9/≥40/≥40/22.4	13.2
#29	USA/69077-1/2019	≥40	14.6	≥40	≥40	≥40	≥40	≥40/≥40/≥40/22.5	12.3
#30	USA/IL/101561-52/2022	≥40	14.6	≥40	≥40	≥40	≥40	≥40/≥40/≥40/22.5	13.2
#31	USA/OK/34563GB/2021	≥40	18.1	≥40	≥40	≥40	≥40	≥40/≥40/≥40/22.4	16.2
#32	USA/IN/57008/2013	≥40	≥40	≥40	≥40	≥40	≥40	≥40/≥40/≥40/22.4	17.0
#33	USA/IN/26125/2012	≥40	22.2	≥40	≥40	≥40	≥40	≥40/≥40/≥40/22.3	11.9
#34	USA/SDSU73	≥40	≥40	≥40	≥40	≥40	≥40	≥40/≥40/≥40/22.4	14.0
#35	USA/IA/24815/2018	≥40	≥40	29.4	≥40	≥40	≥40	≥40/30.6/≥40/22.4	27.1
#36	USA/IA/70388B/2018	≥40	≥40	≥40	≥40	≥40	≥40	≥40/≥40/≥40/22.4	13.2
#37	USA/IA/93743-1/2020	≥40	≥40	≥40	≥40	≥40	≥40	≥40/≥40/≥40/22.4	14.9
#38	USA/UT/88120-4/2019	≥40	≥40	17.0	≥40	≥40	≥40	≥40/17.3/≥40/22.4	14.8
#39	USA/UT/76106-4/2021	≥40	≥40	27.1	≥40	≥40	≥40	≥40/27.1/≥40/22.3	24.1
#40	USA/UT/64317GA/2021	≥40	≥40	15.8	≥40	≥40	≥40	≥40/15.4/≥40/22.5	12.1
#41	USA/UT/18316GA/2021	≥40	≥40	21.5	≥40	≥40	≥40	≥40/21.5/≥40/22.3	17.3
#42	USA/UT/34926-1/2021	≥40	≥40	25.7	≥40	≥40	≥40	≥40/25.5/≥40/22.3	16.9
#43	Mexico/22470-1/2019	≥40	≥40	≥40	≥40	≥40	≥40	≥40/≥40/≥40/22.4	15.2
#44	USA/16572/2017	≥40	≥40	≥40	≥40	≥40	≥40	≥40/≥40/≥40/22.4	17.6
#45	USA/KY/71236GA/2020	≥40	≥40	≥40	≥40	≥40	≥40	≥40/≥40/≥40/22.5	23.1

Red-colored font: samples with discrepant results between ORF5 sequencing and vaccine-like PCR. The samples shown in blue background color in Table 9 are shown with the same background color here.

**Table 11:** Summary of recombination breakpoints of 4 PRRSV-2 isolates.

Isolate#, Name, and Genome Length	nsp2 Genomic Positions	ORF5 Genomic Positions	Breakpoint Positions and Sequence Characteristics
#36 USA/IA/70388B/2018 (14,987 nucleotides)	1316–4510	13,372–13,974	nt 29–6177: wild-type (79.71% nt identity to the Fostera vaccine virus)
			nt 6178–14,987: Fostera vaccine-like (99.64% nt identity to the Fostera vaccine virus)
#37 USA/IA/93743-1/2020 (15,011 nucleotides)	1340–4534	13,396–13,998	nt 1–4853: wild-type (80.87% nt identity to the Fostera vaccine virus)
			nt 4854–9074: Fostera vaccine-like (99.88% nt identity to the Fostera vaccine virus)
			nt 9075–10,541: wild-type (86.77% nt identity to the Fostera vaccine virus)
			nt 10,542–15,011: Fostera vaccine-derived (96.36% nt identity to the Fostera vaccine virus)
#14 USA/AZ/74959GA/2020 (15,386 nucleotides)	1340–4909	13,771–14,373	nt 1–684: wild-type (91.66% nt identity to the Fostera vaccine virus)
			nt 685–12,189: Fostera vaccine-derived (97.34% nt identity to the Fostera vaccine virus)
			nt 12,190–15,386: wild-type (90.24% nt identity to the Fostera vaccine virus)
#12 USA/IN/04584GF/2022 (14,970 nucleotides)	1338–4529	13,391–13,993	nt 1–13,485: wild-type (82.76% nt identity to the Prevacent vaccine virus)
			nt 13,486–14,970: Prevacent vaccine-like (98.18% nt identity to the Prevacent vaccine virus

nt: nucleotide. Recombination of isolate #36 was also previously described by Wang et al. [21].

## Data Availability

The raw data used in this study are available and can be provided upon request.
